# Relative Mortality of the States

**Published:** 1852-03

**Authors:** 


### RELATIVE MORTALITY OF THE STATES.

We have seen it stated that the proportion of deaths to the population in the several States of the Union, as shown by the census of 1850, is as follows: Vermont, 1 in 100; Iowa, 1 in 94; Georgia, 1 in 91; Michigan, 1 in 87; Tennessee, 1 in 86; North Carolina and Alabama, 1 in 85; South Carolina, 1 in 83; Maine, 1 in 77; New Jersey, 1 in 75; Virginia, 1 in 76; Illinois and Delaware, 1 in 73; Arkansas, 1 in 70; Texas, 1 in 69; Rhode Island, 1 in 56; Kentucky and Connecticut, 1 in 64; Maryland, 1 in 60; and Massachusetts 1 in 51.

Some of these results will excite general surprise, and their accuracy indeed seems to us more than questionable. Tennessee, for example, appears to be far more healthy than Kentucky, with a climate, soil, and population as nearly as could be identical. It may be a fact, but we cannot see any reason for it. The mortality of Massachusetts seems to exceed that of any other State, which is probably attributable, in great part, to consumption, but partly also, it is probable, to the greater fullness and accuracy of the vital statistics of that ancient Commonwealth.

